# Leafflower–leafflower moth mutualism in the Neotropics: Successful transoceanic dispersal from the Old World to the New World by actively-pollinating leafflower moths

**DOI:** 10.1371/journal.pone.0210727

**Published:** 2019-01-30

**Authors:** Atsushi Kawakita, Akira A. Wong Sato, Juana R. Llacsahuanga Salazar, Makoto Kato

**Affiliations:** 1 The Botanical Gardens, Graduate School of Science, The University of Tokyo, Tokyo, Japan; 2 Faculty of Forestry Sciences, National Agrarian University La Molina, Lima, Peru; 3 Graduate School of Human and Environmental Studies, Kyoto University, Kyoto, Japan; Swedish University of Agricultural Sciences, SWEDEN

## Abstract

In the Old World tropics, several hundred species of leafflowers (*Phyllanthus* sensu lato; Phyllanthaceae) are engaged in obligate mutualisms with species-specific leafflower moths (*Epicephala*; Gracillariidae) whose adults actively pollinate flowers and larvae consume the resulting seeds. Considerable diversity of *Phyllanthus* also exists in the New World, but whether any New World *Phyllanthus* is pollinated by *Epicephala* is unknown. We studied the pollination biology of four woody *Phyllanthus* species occurring in Peru over a period of four years, and found that each species is associated with a species-specific, seed-eating *Epicephala* moth, here described as new species. Another *Epicephala* species found associated with herbaceous *Phyllanthus* is also described. This is the first description of *Epicephala* from the New World. Field-collected female moths of the four *Epicephala* species associated with woody *Phyllanthus* all carried pollen on the proboscises, and active pollination behavior was observed in at least two species. Thus, *Epicephala* moths also pollinate New World *Phyllanthus*. However, not all of these *Epicephala* species may be mutualistic with their hosts, because we occasionally observed females laying eggs in developing fruits without pollinating. Also, the flowers of some *Phyllanthus* species were visited by pollen-bearing thrips or gall midges, which potentially acted as co-pollinators or primary pollinators. Phylogenetic analysis showed that the New World *Epicephala* associated with woody *Phyllanthus* are nested within lineages of Old World active pollinators. Thus, actively-pollinating *Epicephala* moths, which originated in the Old World, successfully colonized the New World probably across the Pacific and established mutualisms with resident *Phyllanthus* species, although whether any of the relationships are obligate requires further study. There is likely a major radiation of *Epicephala* still to be found in the New World.

## Introduction

Obligate pollination mutualism between plants and actively pollinating, seed parasitic pollinators represent some of the most sophisticated examples of plant–pollinator coevolution [1]. Examples include the fig–fig wasp [2,3], yucca–yucca moth [4], and leafflower–leafflower moth mutualisms [5,6], wherein the plants sacrifice a subset of the seeds as nourishment of pollinator larvae in return for pollination services. The pollinating insects have evolved to actively pollinate host flowers to ensure food (developing seeds) for their larvae, and have morphological features that enhance active pollination, such as the coxal comb and pollen pockets in fig wasps [7], maxillary tentacles in yucca moths [8], and hairy proboscis in leafflower moths [9]. Usually a subset of the seeds is left uneaten by the pollinator larvae, providing net benefit of the mutualism for the plants. Plant specializations to these pollinators have led to highly restrictive floral structures and/or loss of nectar reward, making their flowers hardly attractive to ordinary flower visitors.

**Fig 1:**
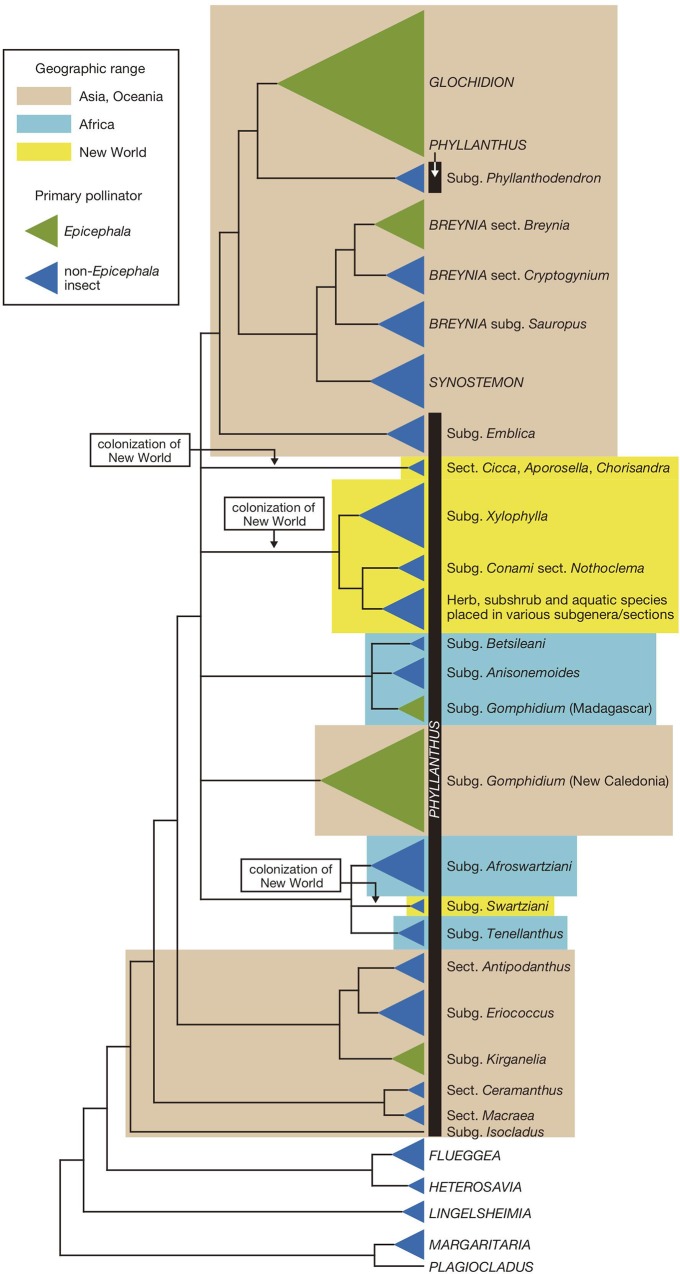
Phylogeny of the tribe Phyllantheae. The tree is based on the most recent molecular phylogenetic analyses of the tribe [12,14]. Species richness of each terminal clade is provided as the area of the clade triangle. The five clades containing *Epicephala* moth-pollinated plants are indicated in green, and the rest is colored blue. Lineages occurring in Asia/Oceania, Africa, and the New World are indicated by beige, turquoise, and yellow boxes, respectively. Although the number of *Phyllanthus* colonizations from the Old World to the New World depends on how the internal polytomy is resolved, suspected three colonization events are indicated on the tree. A group of entirely Madagascan species was recently placed in subgenus *Gomphidium* [27] and thus is tentatively labeled “Subg. *Gomphidium* (Madagascar)”, but note that they are distinct from true *Gomphidium* in New Caledonia. Figure modified from [11].

Leafflowers are the plants in the genera *Glochidion*, *Breynia*, and *Phyllanthus* in the tribe Phyllantheae (Phyllanthaceae), comprising about 1,200 species distributed throughout the Old World and New World tropics [10,11]. The mutualism with leafflower moths, or moths in the genus *Epicephala* (Gracilariidae), was initially discovered in three species of *Glochidion* in Japan [5], but later studies showed that as many as 500 leafflower species, occurring throughout tropical Asia, Africa, Australia, and the Pacific, are mutualistic with species-specific, actively pollinating *Epicephala* moths [12]. These plants bear small (up to 3–4 mm), greenish, unisexual flowers that are visited nocturnally by the females of *Epicephala* moths. The moth uses the hairy proboscis to actively collect pollen on male flower and deposit pollen on the stigma, after which it lays an egg in the flower that it has just pollinated. In most leafflower species, the number of seeds per fruit is 6 (but can be up to 20 in some *Glochidion*) [11]. The proportion of the seeds in each fruit consumed by single *Epicephala* larva is variable among species, but a subset of the seeds usually remains intact after moth consumption [13]. Phylogenetic analysis of the plants and the moths indicated that, whereas the active pollination behavior originated only once in *Epicephala*, specialization to *Epicephala* pollination occurred independently in at least five distinct leafflower lineages (Fig 1) [12].

While knowledge on the diversity and evolution of the leafflower–moth association is accumulating in the Old World tropics, virtually nothing is known about the pollination biology of leafflowers in the New World, despite the fact that there are ca. 250 *Phyllanthus* species throughout the New World. Cuba and Venezuala harbor particularly high diversity with 50 and 58 species, respectively [10]. Molecular phylogenetic analysis suggested that New World *Phyllanthus* group to into three clades of entirely New World species (Fig 1) [14]. These clades occupy derived positions on the phylogeny (Fig 1), indicating that *Phyllanthus* plants colonized the New World from the Old World multiple times. Based on divergence time estimation using fossil calibrations [12], New World colonization by *Phyllanthus* occurred no earlier than the Oligocene (33.9–23.0 Ma). Although the transboreal tropical forest spanning the northern continental area during warm periods of the late Paleocene and early Eocene (ca. 50–52 Ma) [15] would presumably have allowed overland dispersal by these tropical plants, *Phyllanthus* colonization of the New World is too young to be explained by such a scenario and thus is likely the result of transoceanic dispersal.

Due to dearth of fossil Gracillariidae, the divergence times of *Epicephala* is much less reliable. However, available estimation based on COI molecular clock suggests that the genus originated around ca. 25 Ma [12]. Thus, the Neotropics was well separated from the Old World tropics by ocean mass by the time actively pollinating *Epicephala* evolved. Although intercontinental dispersal by tropical insects such as *Epicephala* seems unlikely, *Epicephala* has repeatedly colonized remote oceanic islands of the Pacific [16], suggesting that intercontinental, transoceanic dispersal may also be feasible. Because presently there are no *Epicephala* species described from the New World, we set out to determine whether *Epicephala* occurs in the New World, and if so, whether they are mutualistic with the host *Phyllanthus*, with an aim to understand the global distribution and diversity of the leafflower–leafflower moth mutualism.

## Materials and methods

### Study sites and materials

The study was conducted in Peru during four field expeditions conducted each year during 2013–2016. The first study site, La Florida (6°52'05"S, 79°07'43"W), is located between 900–1,200 m a.s.l. on the western slope of the Andes Mountains and harbors a seasonally dry tropical forest. Three *Phyllanthus* species, *P*. *salviifolius*, *P*. *graveolens*, and *P*. *huallagensis*, were studied at La Florida. Fieldwork was conducted during 6–7 December 2013, 29–31 October 2014, 16–18 November 2015, and 24–26 August 2016. The second site, Tarapoto (6°28'08"S, 76°21'13"W), is located on the Amazonian (eastern) side of the Andes Mountains at around 350 m a.s.l. and possesses a wet tropical forest. *Phyllanthus acuminatus* was studied at Tarapoto during 26–28 November 2013, 20–23 October 2014, and 21–22 November 2015. In addition to the above four woody *Phyllanthus*, three herbaceous *Phyllanthus* species were encountered during the course of the study; *P*. *stipulatus* and *P*. *orbiculatus* at Tarapoto, and *P*. *amarus* at a lowland Amazonian site at Iquitos (3°34'00"S, 73°07'11"W; ca. 100 m a.s.l.) visited during 29 August–2 September 2016. Pollination by *Epicephala* has never been found in herbaceous *Phyllanthus* in the Old World. However, because several herbaceous *Phyllanthus* species in Asia are associated with *Epicephala* that lack pollination behavior [12], the above three herbaceous species were also included in the study.

*Phyllanthus salviifolius* and *P*. *huallagensis* are members of the subgenus *Xylophylla* [14], a New World endemic group of ca. 90 species with its center of diversity in the Caribbean. *Phyllanthus salviifolius* belongs to the section *Oxalistylis* and is distributed in Costa Rica, Colombia, Venezuela, Ecuador, and Peru, whereas *P*. *huallagensis* belongs to the section *Elutanthos* and is only known from Peru [10]. *Phyllanthus acuminatus* and *P*. *graveolens* belong to the New World section *Nothoclema* of the subgenus *Conami*, which contains 10 species and ranges from Mexico to Argentina [17]. Both species are widely distributed from Mexico to the north to Peru (*P*. *graveolens*) and Argentina (*P*. *acuminatus*) to the south. Although *Xylophylla* and *Nothoclema* are not sister taxa, they group with other Neotropical herb, subshrub, and aquatic species and form the largest New World *Phyllanthus* radiation (Fig 1). Of the three herbaceous species studied, *P*. *orbiculatus* (sect. *Apolepis*) belongs to the same subgenus *Conami* as *Nothoclema* [18] and thus is likely a part of this large New World clade. On the other hand, *P*. *amarus* belong to the distantly related subgenus *Swartziani* [19], which represents an independent New World colonization (Fig 1). The phylogenetic position of *P*. *stipulatus* is presently uncertain.

**Fig 2:**
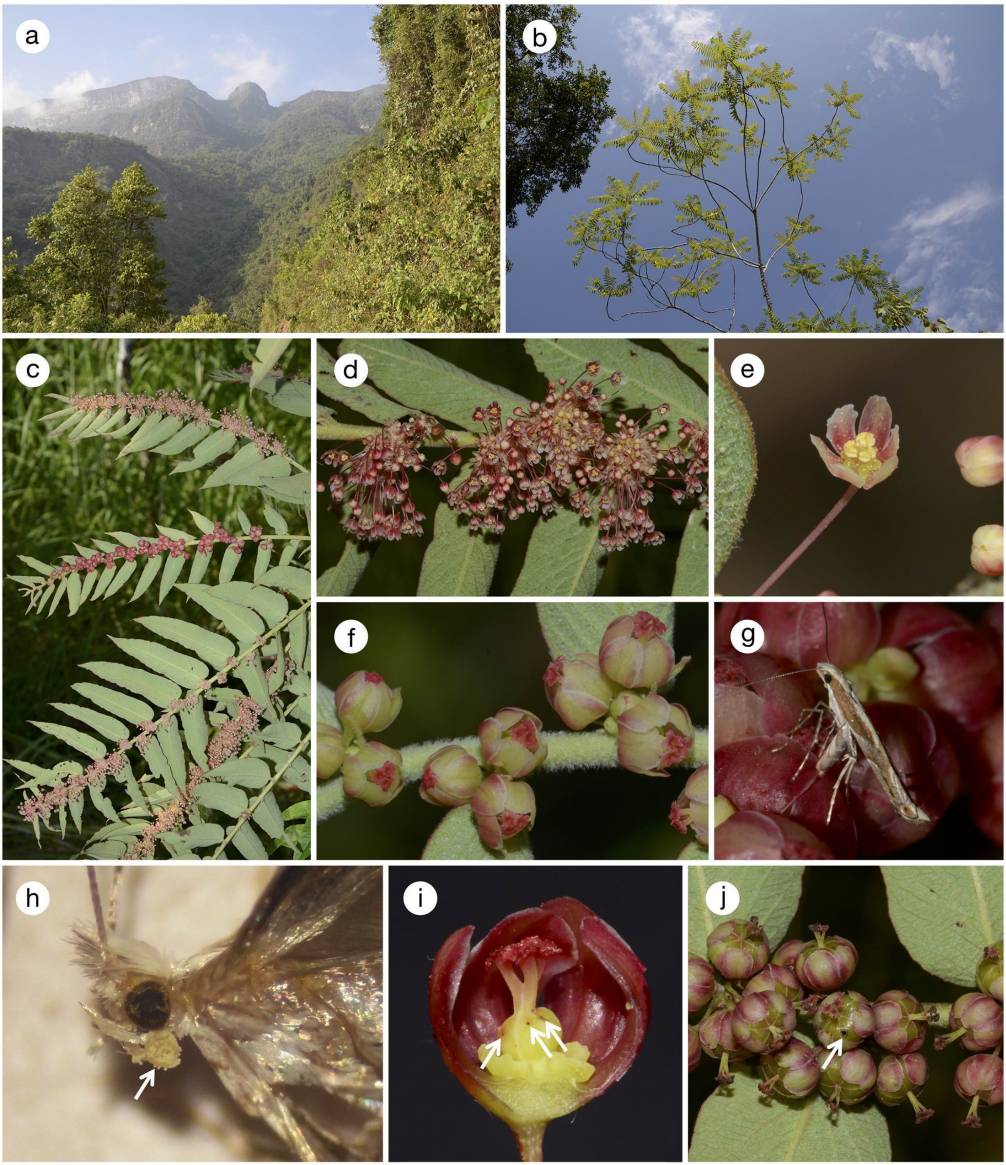
*Phyllanthus salviifolius* and associated *Epicephala*. (a) Habitat of *P*. *salviifolius* at La Florida; (b) habit; (c) branches bearing male and female flowers; (d) male flowers; (e) close-up of a male flower; (f) female flowers; (g) an *E*. *anomala* female ovipositing in ovary by penetrating the tepal with the ovipositor; (h) proboscis of field-collected female *E*. *anomala* (arrow) covered with *Phyllanthus* pollen; (i) a young fruit with *Epicephala* oviposition scars (arrows). Two front tepals have been artificially removed; (j) developed fruits, one of which having an exit hole (arrow) excavated by *Epicephala* larva.

**Fig 3:**
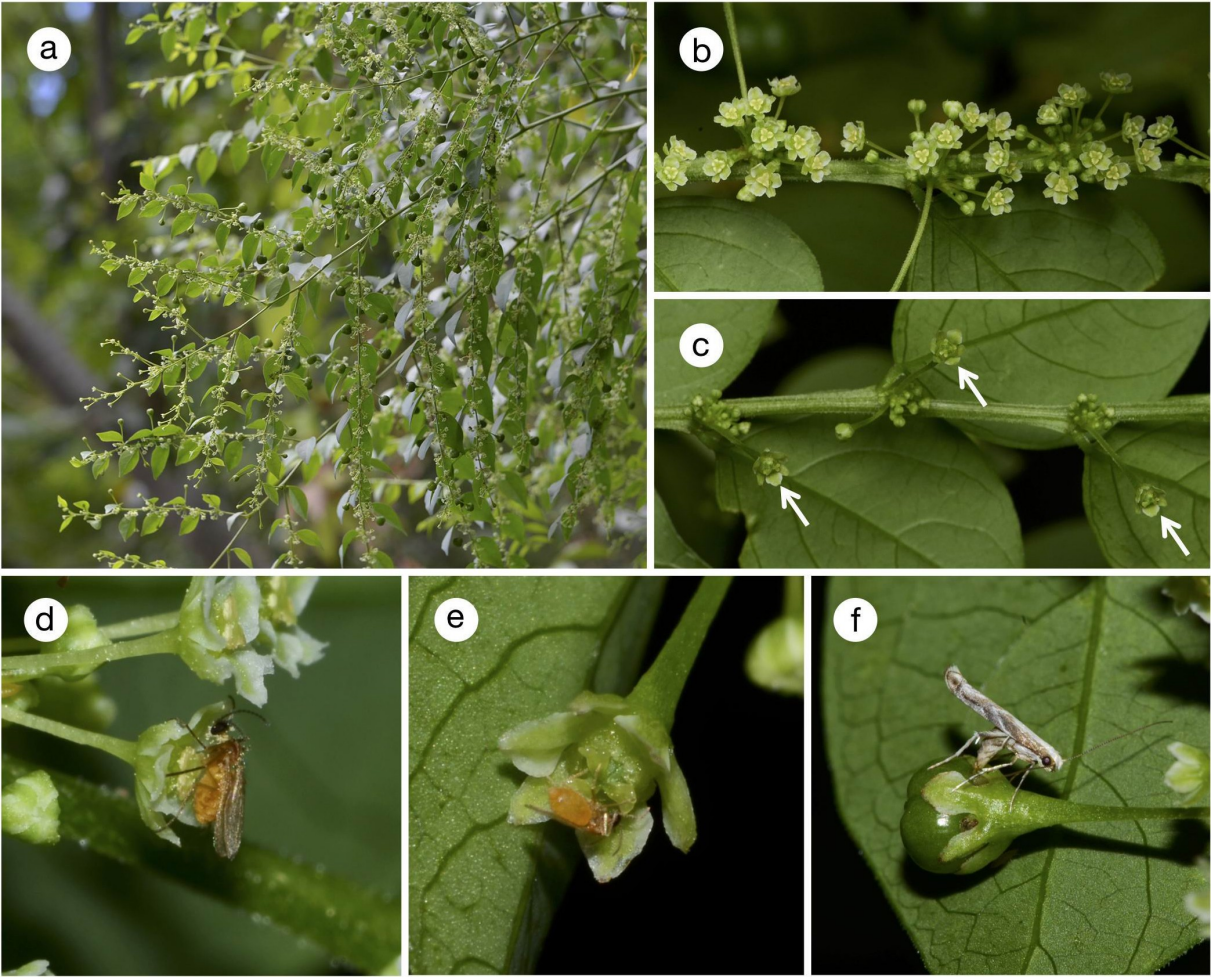
*Phyllanthus acuminatus* and associated insects. (a) Habit of *P*. *acuminatus*; (b) male flowers; (c) female flowers (arrows); (d) male flower visited by a gall midge. Note that the head and thorax of the insect is dusted with pollen; (e) female flower visited by a gall midge, which is presumably consuming nectar; (f) an *E*. *acuminatella* female ovipositing in young fruit.

**Fig 4:**
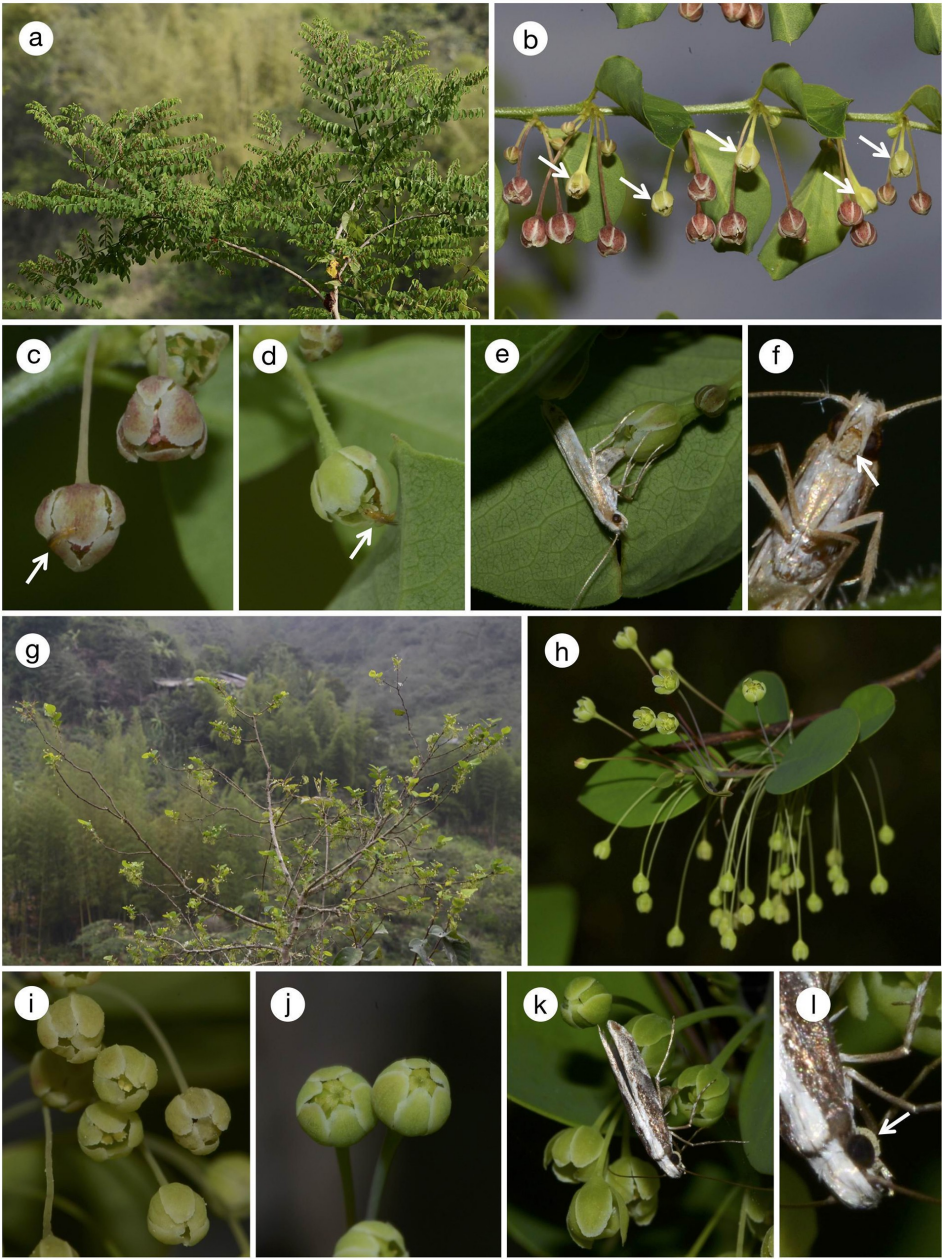
*Phyllanthus graveolens*, *P*. *huallagensis*, and associated insects. (a)–(f) *P*. *graveolens*; (g)–(l) *P*. *huallagensis*. (a) Habit; (b) a branch bearing male and female flowers. Female flowers are indicated by arrows, and the rest are all male flowers; (c) male flower visited by a thrips (arrow); (d) female flower visited by a thrips (arrow); (e) *Epicephala graveolensella* female ovipositing in female flower; (f) proboscis of *E*. *graveolensella* female (arrow) covered with pollen; (g) habit; (h) a branch bearing male flowers below the leaves and erect, pollinated female flowers above the leaves; (i) close-up of male flowers; (j) close-up of female flowers; (k) *E*. *huallagensiella* ovipositing in female flower; (l) proboscis of *E*. *graveolensella* female (arrow) bearing pollen.

All the four woody *Phyllanthus* species are found on forest edges or on disturbed land with abundant sunlight. Only *P*. *salviifolius* is a tree that reaches up to 10 m (Fig 2), whereas the other three species are shrubs of less than 3 m (Figs 3 and 4). *Phyllanthus salviifolius* also differs from the other three species in that a single flowering branch either produces male or female flowers (Fig 2C). The male flowers of *P*. *salviifolius* possess long pedicels and as many as 30 flowers aggregate on each axil (Fig 2D and 2E). The female flowers in turn are hardly pedicellate, and the tepals form a globular structure that covers the ovary and the style, leaving only the stigmatic surface exposed (Fig 2F). By contrast, both male and female flowers of *P*. *acuminatus* have spread tepals and exposed anthers and styles (Fig 3B and 3C). The flowers of *P*. *graveolens* and *P*. *huallagensis*, although belonging to different subgenera, resemble each other in that the tepals of both male and female flowers form a globe and almost entirely covers the anthers and the styles (Fig 4B, 4I and 4J). Once pollinated, the female flowers of *P*. *huallagensis* become erect until they mature into fruits and disperse the seeds (Fig 4H). Nectary discs are present in both male and female flowers of all four species.

**Fig 5:**
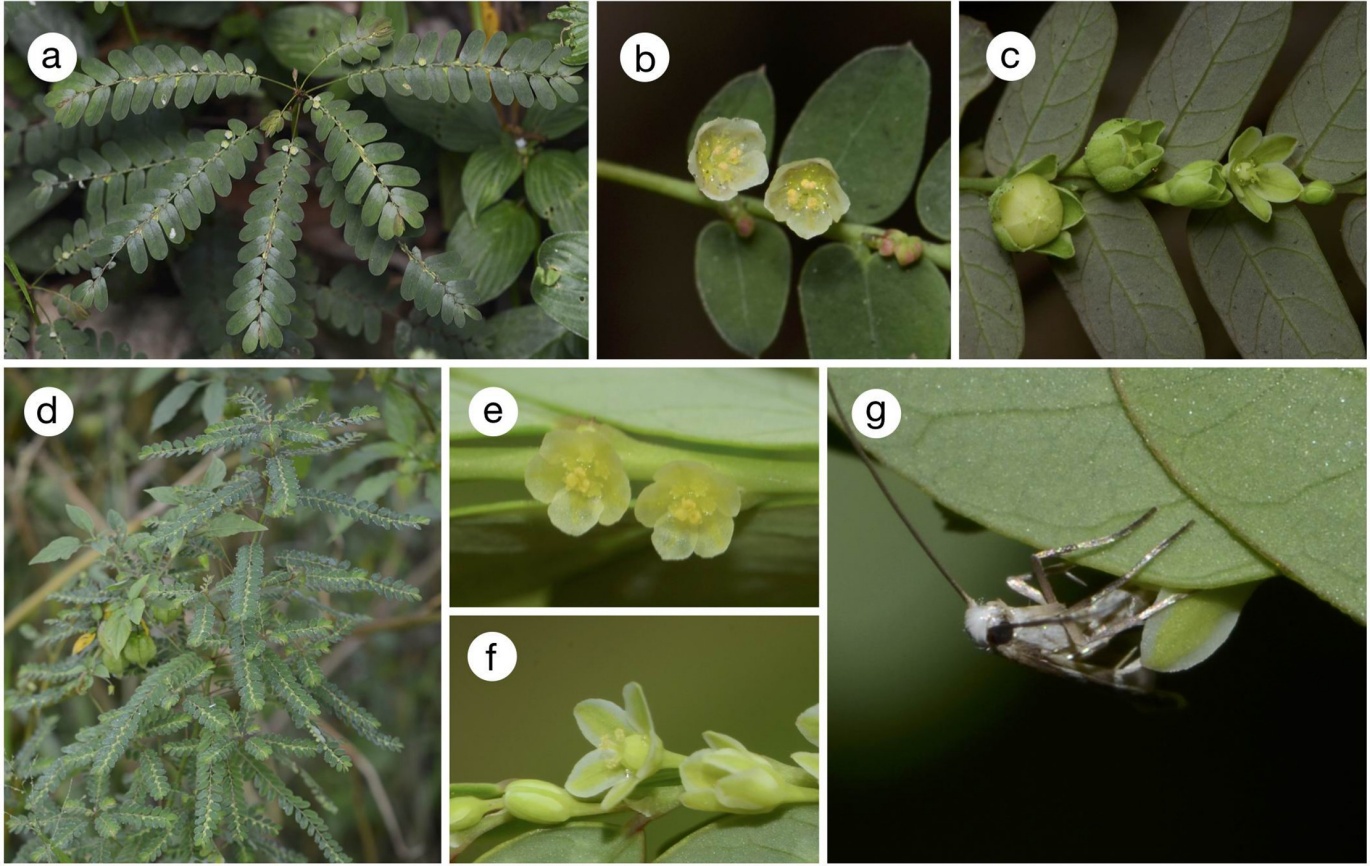
*Phyllanthus stipulatus*, *P*. *amarus*, and associated *Epicephala*. (a)–(c) *P*. *stipulatus*; (d)–(g) *P*. *amarus*. (a) Habit; (b) male flowers; (c) female flower and young fruits; (d) habit; (e) male flowers; (f) female flower; (g) *Epicephala chancapiedra* female ovipositing in young fruit.

The three herbaceous *Phyllanthus* species are common in sunny habitats along roadsides or on disturbed land and bear small, nectariferous male and female flowers (Fig 5)

### Sampling and species delimitation of *Epicephala* moths

To determine whether the studied *Phyllanthus* species are associated with *Epicephala*, we initially inspected the fruits and the nearby foliage for the presence of *Epicephala* larvae and pupae. Because *Epicephala* moths were found in all the studied *Phyllanthus*, we sampled the fruits haphazardly in the field and incubated them at room temperature in plastic containers to rear the larvae. Adults that emerged were used for morphological examination and sequencing of the insect barcoding region to delimit species and assess host specificity. Morphological examinations follow standard dissection protocol detailed in [20]. In total, 91 adult pinned specimens were examined for the study, from which 28 genital dissections were made. The sensilla on the proboscis of females were also examined because they are an adaptation to facilitate active pollination and are reduced or lost in species that secondarily lost the pollination behavior [9]. Genomic DNA was extracted from 19 of the 28 specimens for which genital dissections were made, and the barcoding region of the cytochrome oxidase subunit I (COI) gene was sequenced using the LCO and HCO primers [21]. DNA extraction, polymerase chain reaction, and sequencing followed the protocols of [22]. The aligned sequence matrix of 612-bp length was subjected to a maximum-likelihood phylogenetic analysis on the IQ-TREE web server [23] using the default settings. Newly obtained sequences have been deposited in DDBJ under accession numbers LC424114–LC424132 and in BOLD under the project EPICE.

### Behavioral observation

The behavior of *Epicephala* moths was studied in the field during 1800–2200 to determine whether any species is an active pollinator. We patrolled plant individuals with flowering branches at night using flashlights, and whenever *Epicephala* moths were found visiting female flower, we recorded whether each moth displayed pollination behavior prior to oviposition and the location of egg deposition. The moths were collected after observation, although some attempts to collect the moths were unsuccessful. The proboscises of the sampled moths were observed under a dissecting microscope for pollen load, and genital dissections were made to identify the species.

In addition, non-*Epicephala* insects that visited the flowers were recorded and captured whenever possible. Each insect specimen was inspected under a dissecting microscope for pollen load to determine whether they could potentially contribute to pollination.

### Pollen and egg loads on female flowers

Although direct observation of pollination behavior provides straightforward evidence that the species is an active pollinator, observation was not sufficient for *Epicephala* associated with *P*. *graveolens* and *P*. *huallagensis*. We therefore examined the female flowers of the two species for pollen and egg loads under a dissecting microscope. If the majority of the pollinated flowers are infested with moth eggs, and unpollinated flowers are free of moth eggs, it is likely that the associated *Epicephala* pollinates before oviposition and acts as the sole pollinator. In turn, if some fraction of pollinated flowers is free of moth eggs, co-pollinator may be present, regardless of whether the associated *Epicephala* actively pollinates. We examined whether there is such an association between pollen and egg loads to compensate for a lack of direct observations in *P*. *graveolens* and *P*. *huallagensis*.

### Phylogenetic positions of New World *Epicephala*

To infer the phylogenetic positions of New World *Epicephala* relative to the Old World species, we analyzed the sequences of the combined mitochondrial COI, nuclear elongation factor 1-alpha (EF1α) and arginine kinase (ArgK) genes. We used the primers and laboratory protocols described in [22] and [24] to obtain EF1αand ArgK sequences for one representative individual of each New World *Epicephala* species, chosen haphazardly from the individuals for which COI was sequenced as described. Obtained sequences were analyzed with published COI, EF1α, and ArgK sequence data for 29 Old World *Epicephala* and related *Conopomorpha flueggella* (associated with *Flueggea*). Sequences of *Cuphodes diospyrosella*, *Stomphastis labyrinthica*, and *Melanocercops ficuvorella*, obtained from the database, were used as outgroups. Phylogenetic analysis was done using IQ-TREE as described above for COI. Newly obtained EF1αand ArgK sequences have been deposited in DDBJ under accession numbers LC424133–LC424142.

### Nomenclatural acts

The electronic edition of this article conforms to the requirements of the amended International Code of Zoological Nomenclature, and hence the new names contained herein are available under that Code from the electronic edition of this article. This published work and the nomenclatural acts it contains have been registered in ZooBank, the online registration system for the ICZN. The ZooBank LSIDs (Life Science Identifiers) can be resolved and the associated information viewed through any standard web browser by appending the LSID to the prefix “http://zoobank.org/”. The LSID for this publication is: urn:lsid:zoobank.org:pub:7B78433F-07E2-40ED-BAE1-C083AF3304C2. The electronic edition of this work was published in a journal with an ISSN, and has been archived and is available from the following digital repositories: PubMed Central, LOCKSS.

## Results

### Species description

**Fig 6:**
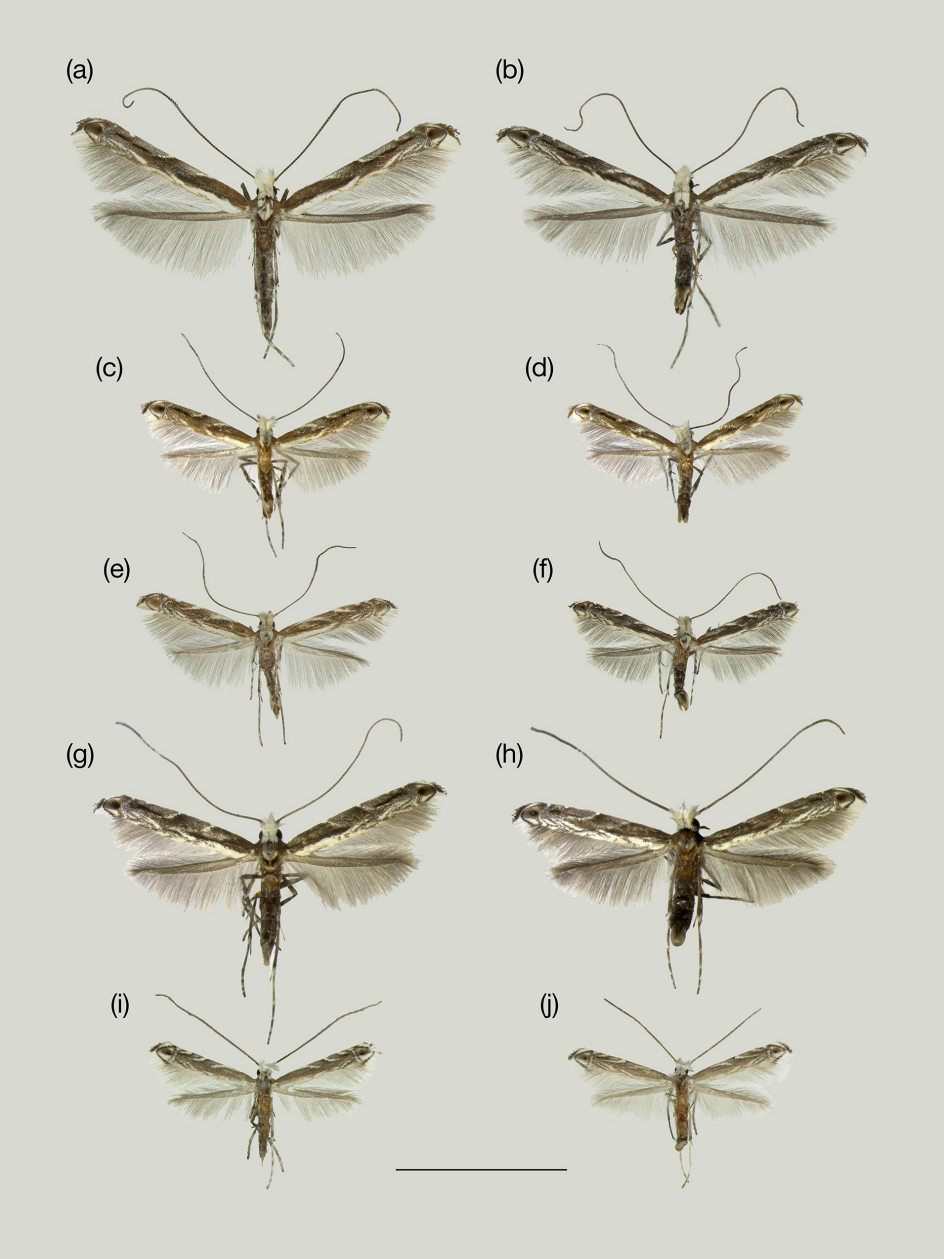
Representative specimens of the five *Epicephala* species in Peru. (a) *E*. *anomala*, ♀ (holotype); (b) *E*. *anomala*, ♂; (c) *E*. *acuminatella*, ♀ (holotype); (d) *E*. *acuminatella*, ♂; (e) *E*. *graveolensella*, ♀ (holotype); (f) *E*. *graveolensella*, ♂; (g) *E*. *huallagensiella*, ♀ (holotype); (h) *E*. *huallagensiella*, ♂; (i) *E*. *chancapiedra*, ♀ (holotype); (j) *E*. *chancapiedra*, ♂. Bar = 5 mm.

Morphological examination of the adults that emerged from the fruits of four woody and three herbaceous *Phyllanthus* resulted in recognition of five *Epicephala* species (Fig 6). Four of them were each specific to one of the four woody *Phyllanthus* hosts, whereas the fifth species was broadly associated with the three herbaceous species. Analysis of the COI sequences confirmed the existence of five distinct clades, corresponding to morphologically recognized species (S1 Fig). Intraspecific pairwise sequence variation was within the range of 0–0.16%, whereas pairwise sequence variation between species exceeded 2.7%.

Below we describe the five *Epicephala* species. Detailed description of morphology is provided in S1 Text.

#### *Epicephala anomala* Kawakita & Kato, sp. nov.

urn:lsid:zoobank.org:act:171DA3E5-2318-4A01-90FD-5684CEA33F83

**Diagnosis.** This species and the following *Epicephala acuminatella* are unlike any other *Epicephala* species in having sclerotized blade on the posterior side of cucullus and elongated inward projection on dorsal margin of cucullus. This species can be clearly distinguished from *E*. *acuminatella* by the broader and overall straight shape of the sclerotized blade on cucullus and distinctly narrower lamella postvaginalis.

**Description.** Morphological description is provided in S1 Text.

**Material examined.** 15♂, 27♀. Holotype ♀–PERU: Cajamarca: La Florida, 1,200 m, collected as larva in fruit of *Phyllanthus salviifolius* and reared to adult, 31.x.2014 (MUSM). Paratypes–same locality as holotype, collected as larvae in fruits of *P*. *salviifolius* and reared to adults, 31.x.2014, 11♂, 21♀ (ZMUT). Other specimens–same locality as holotype, collected as larvae in fruits of *P*. *salviifolius* and reared to adults, 7.xii.2013, 4♂, 5♀.

**DNA barcodes.** LC424117, LC424120, LC424121.

**Known host.** Known only from *Phyllanthus salviifolius*. Larva feeds on the seeds.

**Distribution.** Known only from the type locality.

**Etymology.** The name *anomala* (an adjective) derives from the Latin *anomalus* (= abnormal, irregular) because this species possesses a perplexing combination of features that are characteristic of active pollinators (active pollen collection) and non-pollinators (lack of sensilla on proboscis, oviposition in young fruit).

#### *Epicephala acuminatella* Kawakita & Kato, sp. nov.

urn:lsid:zoobank.org:act:094F1643-22F3-414B-8A82-4AFABBB1DF1F

**Diagnosis.** This species is morphologically similar to *E*. *anomala* but differs from the latter in having distinctly curved sclerotized blade on cucullus and wider lamella postvaginalis.

**Description.** Morphological description is provided in S1 Text.

**Material examined.** 3♂, 8♀. Holotype ♀–PERU: San Martín: Tarapoto, 350 m, collected as larva in fruit of *Phyllanthus acuminatus* and reared to adult, 23.xi.2015 (MUSM). Paratypes–same locality as holotype, collected as larvae in fruits of *P*. *acuminatus* and reared to adults, 23.xi.2015, 3♂, 7♀ (ZMUT).

**DNA barcodes.** LC424114, LC424116, LC424126, LC424127.

**Known host.** Known only from *Phyllanthus acuminatus*. Larva feeds on the seeds.

**Distribution.** Known only from the type locality.

**Etymology.** The name *acuminatella* (an adjective) derives from the species name of the host plant *P*. *acuminatus*.

#### *Epicephala graveolensella* Kawakita & Kato, sp. nov.

urn:lsid:zoobank.org:act:B0FB196B-8F57-43AF-B154-B9C9AAEA3165

**Diagnosis.** This species is distinct from all other *Epicephala* species in having dense sclerotized spines on inner surface of cucullus and sclerotized, hook-like projection at the base of ventral margin of cucullus. Slender cornutus penetrating over half the length of aedeagus is also not found in any other *Epicephala* species.

**Description.** Morphological description is provided in S1 Text.

**Material examined.** 3♂, 3♀. Holotype ♀–PERU: Cajamarca: La Florida, 900 m, collected as larva in fruit of *Phyllanthus graveolens* and reared to adult, 31.x.2014 (MUSM). Paratypes–same locality as holotype, collected as larvae in fruits of *P*. *graveolens* and reared to adults, 31.x.2014, 3♂, 2♀ (ZMUT).

**DNA barcodes.** LC424118, LC424122, LC424123.

**Known host.** Known only from *Phyllanthus graveolens*. Larva feeds on the seeds.

**Distribution.** Known only from the type locality.

**Etymology.** The name *graveolensella* (an adjective) derives from the species name of the host plant *P*. *graveolens*.

#### *Epicephala huallagensiella* Kawakita & Kato, sp. nov.

urn:lsid:zoobank.org:act:FA341B61-8BFC-48CC-B575-3EDA66E078C5

**Diagnosis.** This species differs from other *Epicephala* species in having ventrally projecting hairs at the base of ventral margin of cucullus and inwardly curled sacculus with acute projection and row of spines on dorsal margin.

**Description.** Morphological description is provided in S1 Text.

**Material examined.** 11♂, 14♀. Holotype ♀–PERU: Cajamarca: La Florida, 900 m, collected as larva in fruit of *Phyllanthus huallagensis* and reared to adult, 18.xi.2015 (MUSM). Paratypes–same locality as holotype, collected as larvae in fruits of *P*. *huallagensis* and reared to adults, 18.xi.2015, 11♂, 13♀ (ZMUT).

**DNA barcodes.** LC424115, LC424119, LC424128, LC424129.

**Known host.** Known only from *Phyllanthus huallagensis*. Larva feeds on the seeds.

**Distribution.** Known only from the type locality.

**Etymology.** The name *huallagensiella* (an adjective) derives from the species name of the host plant *P*. *huallagensis*.

#### *Epicephala chancapiedra* Kawakita & Kato, sp. nov.

urn:lsid:zoobank.org:act:DC072AC2-4EA5-4F17-92D5-06D91A99B1D0

**Diagnosis.** This species is morphologically similar to *E*. *nudilingua* in having forked lamella postvaginalis but differs from the latter in lacking well developed cornutus and more rectangular sacculus.

**Description.** Morphological description is provided in S1 Text.

**Material examined.** 5♂, 2♀. Holotype ♀–PERU: San Martín: Tarapoto, 350 m, collected as larva in fruit of *Phyllanthus stipulatus* and reared to adult, 26.xi.2013 (MUSM). Paratypes–same locality as holotype, collected as larvae in fruits of *P*. *stipulatus* and reared to adults, 26.xi.2013, 3♂, 1♀ (ZMUT); Other specimens–same locality as holotype, collected as larva in fruit of *P*. *orbiculatus* and reared to adult, 29.xi.2013, 1♀; PERU: Loreto: Iquitos, 100 m, collected as larva in fruit of *P*. *amarus* and reared to adult, 2.ix.2016, 1♀.

**DNA barcodes.** LC424124, LC424125, LC424130–LC424132.

**Known host.** *Phyllanthus stipulatus*, *P*. *orbiculatus*, and *P*. *amarus*. Larva feeds on the seeds.

**Distribution.** Amazonian Peru (San Martín, Loreto).

**Etymology.** The name *chancapiedra* (a noun in apposition) derives from the Incan name for herbaceous *Phyllanthus* ‘chanca piedra’ (stone breaker). Herbaceous *Phyllanthus* such as *P*. *amarus* are locally used as a medical herb to remove kidney stones.

### Adult *Epicephala* behavior and other floral visitors

The flowers of the four woody *Phyllanthus* species were not always available during our fieldwork, so the number of occasions on which we encountered adult *Epicephala* was limited. Nevertheless, we were able to successfully observe the behavior of three moths on each of *P*. *salviifolius* and *P*. *acuminatus*, one moth on *P*. *graveolens*, and two moths on *P*. *amarus*. We also observed one moth in the act of oviposition on *P*. *huallagensis*, but whether this moth pollinated the flower prior to oviposition is unclear. Below we provide details of the behavior of each moth species. A summary of the results is given in Table 1.

**Table 1:** Host plant species and pollinator habit of the five *Epicephala* species described in this study.

*Epicephala* moth species	Host plant species	Adult behavior observed (individuals)	Pollination behavior	Pollen load on proboscis	Oviposition site	Proboscis sensilla
*E*. *anomala*	*P*. *salviifolius*	3	present (observed only once in >20 oviposition events)	present	young fruit	absent
*E*. *acuminatella*	*P*. *acuminatus*	3	absent	present	young fruit	present
*E*. *graveolensella*	*P*. *graveolens*	1	present	present	flower	present
*E*. *huallagensiella*	*P*. *huallagensis*	1	present^a^	present	flower	present
*E*. *chancapiedra*	*P*. *stipulatus*, *P*. *orbiculatus*, *P*. *amarus*	2	absent	absent	young fruit	absent

^a^Pollination behavior was not directly observed in this species and is based on the presence of pollen on the stigma and an egg in the ovary of the female flower visited by this species. See text for details.

We encountered the flowering of *P*. *salviifolius* once in October 2014. There were numerous male flowers, but many female flowers had already been pollinated and started to develop into fruits. We observed three female individuals of *E*. *anomala* visiting such flowers with developing ovaries. After finding suitable female flower in which to oviposit, the moths bended the abdomen and inserted the ovipositor into the ovary through the interspace between the tepals or by penetrating the tepals directly with the ovipositor, without exhibiting pollination behavior (Fig 2G; S1 Video). We observed more than 10 such oviposition events in each of two moths and three such ovipositions in one moth. Perplexingly, however, after capturing the three moths and inspecting their proboscises under a microscope, we found that their proboscises were coated with *Phyllanthus* pollen in a manner very similar to the proboscises of field-collected individuals of actively pollinating species. Thus, they have apparently visited the male flower before visiting female flowers and actively collected pollen. In addition, we observed one moth stretching the proboscis and inserting it into the flower several times before oviposition, which we regard as active pollination (S2 Video). This was observed in only one of >10 oviposition events by this moth, so active pollination occurred only occasionally in *E*. *anomala*. No other flower visitors were observed except one thrips individual that was found on male flower during the daytime.

The behavior of adult *E*. *acuminatella* on *P*. *acuminatus* was observed three times; twice in 2013 and once in 2014. Oviposition was observed twice in one moth and once each in the other two moths. On all occasions, the moths laid eggs in young fruits, but not female flowers, without exhibiting pollination behavior (Fig 3F). However, the two moths that were successfully captured both had their proboscises coated with *Phyllanthus* pollen, indicating that they had actively collected pollen before visiting female flowers. We collected one additional female that was resting on *P*. *acuminatus* foliage, and this individual also had pollen on the proboscis. Both male and female flowers of *P*. *acuminatus* were visited frequently by gall midges in the evening (Fig 3D and 3E). They pushed their mouthparts against the floral discs presumably to take in nectar and had many *Phyllanthus* pollen grains attached to their bodies (Fig 3D). No other flower visitors were found either during the daytime or in the evening. Gall midges were found resting on flowers or leaves of *P*. *acuminatus* during the daytime.

We studied the flower of *P*. *graveolens* in November 2015 and observed one adult *Epicephala* on female flower. Unlike *E*. *anomala* or *E*. *acuminatella* that laid eggs in young fruits, this *Epicephala* visited female flower, pollinated the stigma with the proboscis and subsequently laid an egg (Fig 4E). Nine pollen grains were attached to the stigma of this flower and one egg was laid internally on the ovary wall. We failed to capture this moth, but inspection of the photograph taken of this moth while resting on the branch shows that the proboscis was dusted with pollen (Fig 4F). Thus, we consider this moth an active pollinator, similar to those typically found on Old World leafflowers. Because the moth was not captured, we could not confirm the species morphologically, but DNA barcoding of the egg indicated that the moth is *E*. *graveolensella*. In addition to *Epicephala*, thrips were commonly found inside both male and female flowers (Fig 4C and 4D). Both juvenile and adult thrips were found, indicating that they use *P*. *graveolens* flowers as brood sites. Pollen grains were attached invariably to 55 adult thrips individuals collected haphazardly on male flowers, and, out of 36 adult thrips individuals sampled on female flowers, two had pollen on their bodies, suggesting that thrips may act as co-pollinators.

On *P*. *huallagensis*, one moth was found in the act of oviposition (Fig 4K), but because this moth was the only individual that we could observe, we were unable to assess whether pollination takes place prior to oviposition. We also failed to capture this moth and thus could not inspect its proboscis microscopically, but the photograph shows that the proboscis is coated with pollen (Fig 4L). Thirty-one pollen grains were attached to the stigma of the flower visited by this moth, and one egg was laid internally on the ovary wall. Using DNA barcode, we confirmed that the moth is *E*. *huellagensiella*. No other flower visitors were found during the observation, although we found two thrips individual, without pollen, in one of 206 female flowers that we dissected.

Finally, two *E*. *chancapiedra* individuals were observed on *P*. *amarus*. Both individuals neither pollinated female flowers nor carried pollen on the proboscises, and laid eggs in young fruits (Fig 5G). Thus, this species is likely a non-pollinating parasite, similar to *Epicephala* associated with herbaceous *Phyllanthus* in the Old World. Ants and stingless bees were frequent visitors to *P*. *amarus* flowers, which likely contributed to pollination.

### Proboscis morphology

**Fig 7:**
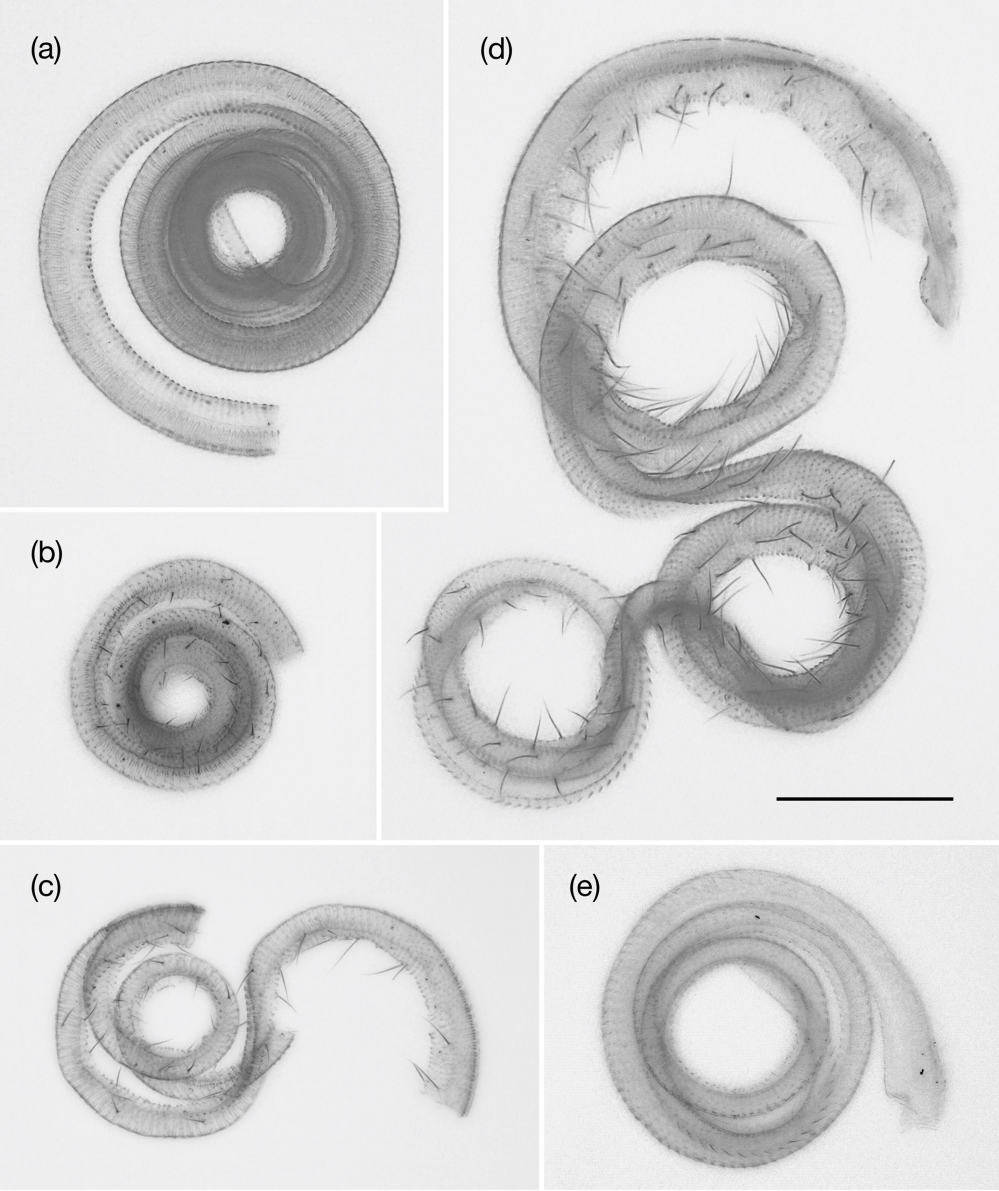
Female proboscis of the five *Epicephala* species in Peru. (a) *E*. *anomala*; (b) *E*. *acuminatella*; (c) *E*. *graveolensella*; (d) *E*. *huallagensiella*; (e) *E*. *chancapiedra*. Because the proboscises were broken during slide preparation, only the distal portions of the proboscises are shown for (b) and (c). Bar = 100 μm, shared for all photographs.

The proboscises of the five *Epicephala* species were observed under a microscope to determine whether sensilla are present. The sensilla were clearly present on the proboscises of *E*. *acuminatella*, *E*. *graveolensella*, and *E*. *huellagensiella*, but absent in those of *E*. *anomala* and *E*. *chancapiedra* (Fig 7).

### Pollen and egg loads on female flowers

Because behavioral observations were not sufficient for *E*. *graveolensella* and *E*. *huallagensiella*, we examined the female flowers of *P*. *graveolens* and *P*. *huallagensis* to determine whether there is association between pollination status and presence of moth eggs. In *P*. *graveolens*, we inspected 173 female flowers, of which 112 were pollinated. Out of the 112 pollinated flowers, only 64 (57.1%) received *Epicephala* eggs (Table 2), indicating that co-pollinators, such as thrips, may be responsible for the pollination of the remaining flowers that had not received moth eggs. An alternative possibility is that *Epicephala* moths pollinated most of the flowers but failed to lay eggs in some of them (loss of eggs subsequent to oviposition is unlikely because moth ovipositions cause visible scars on ovaries which were not observed on flowers without eggs). We also examined 206 female flowers of *P*. *huallagensis* and found 154 that were pollinated. Of these, 130 (84.4%) had moth eggs (Table 2), so the contribution of co-pollinators, if any, is smaller than it is in *P*. *graveolens*. No moth egg was deposited on 61 and 52 unpollinated flowers of *P*. *graveolens* and *P*. *huallagensis* (Table 2).

**Table 2:** Number of flowers with and without *Epicephala* moth eggs among pollinated and unpollinated flowers of *Phyllanthus*.

*Phyllanthus* species	Pollination status	Flowers with egg	Flowers without egg
*P*. *graveolens*	Pollinated	64	48
	Unpollinated	0	61
*P*. *huallagensis*	Pollinated	130	24
	Unpollinated	0	52

### Phylogenetic position of New World *Epicephala*

**Fig 8:**
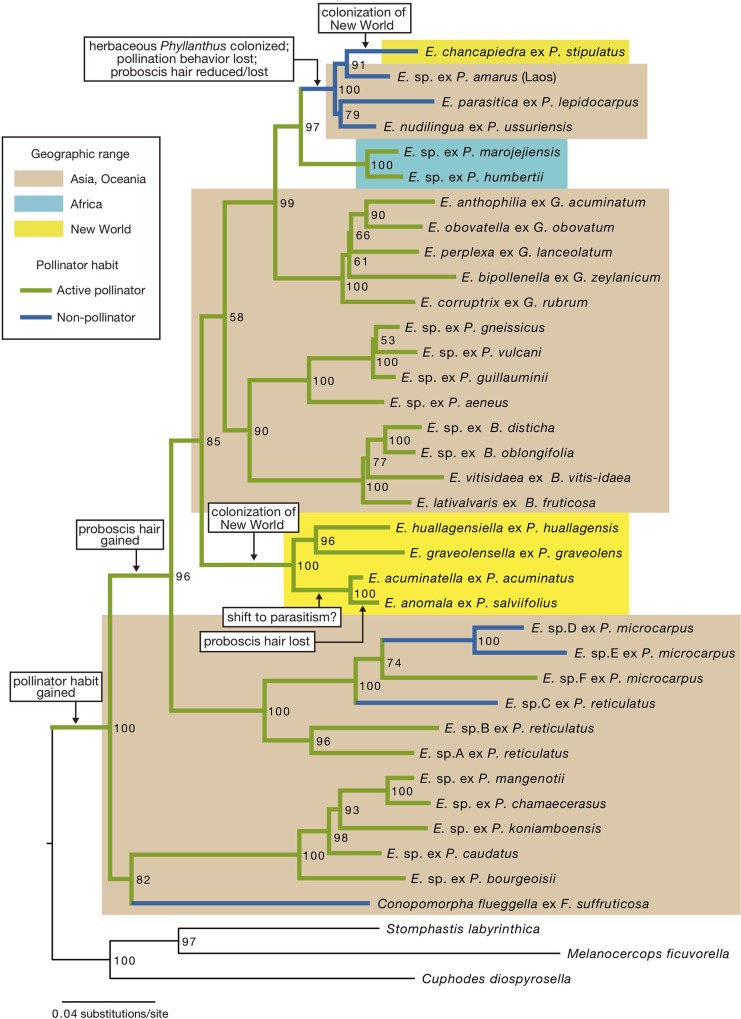
Maximum likelihood phylogeny of the genus *Epicephala* reconstructed based on analysis of combined mitochondrial cytochrome oxidase subunit I (COI), nuclear elongation factor 1-alpha (EF1α) and arginine kinase (ArgK) gene sequences. Blue lines indicate lineages with parasitic lifestyles, including species that either entirely lack the pollination behavior or show pollination behavior only occasionally [9, 12]; green lines indicate lineages with active pollination behavior. Lineages occurring in Asia/Oceania, Africa, and the New World are indicated by beige, turquoise, and yellow boxes, respectively. Each terminal label includes information on host plant species. Abbreviations are: *E*., *Epicephala*; *P*., *Phyllanthus*; *G*., *Glochidion*; *B*., *Breynia*; *F*., *Flueggea*. Numbers at nodes are bootstrap probability values based on 1,000 replications. Major evolutionary events are indicated for selected nodes. *Epicephala anomala* and *E*. *acuminatella* are conservatively labeled as active pollinators, but there is a possibility that they are parasitic (see text for discussion).

Maximum-likelihood analysis of the combined COI + EF1α+ ArgK gene sequences indicated that the four *Epicephala* species associated with woody *Phyllanthus* form a well-supported clade, and this clade is embedded among lineages of Old World active pollinators (Fig 8). In turn, *E*. *chancapiedra* grouped with Old World *Epicephala* species associated with herbaceous *Phyllanthus* (Fig 8). Because Old World *Epicephala* on herbaceous *Phyllanthus* all lack pollination behavior, the ancestor of *E*. *chancapiedra* likely did not possess the pollination behavior by the time it colonized the New World.

## Discussion

Although the presence of *Epicephala* in the New World has been implicated based on the presence of *Epicephala*-like larvae and pupal cocoons on herbarium specimens of Neotropical *Phyllanthus* [25], this is the first report of any *Epicephala* from the New World. The diversity of *Phyllanthus* in Peru is not high as compared to other regions in the Neotropics; however, we found species-specific *Epicephala* species from each of four woody *Phyllanthus* that were studied and one *Epicephala* species associated with three herbaceous *Phyllanthus*. Considering that there are ca. 250 *Phyllanthus* species throughout the New World, there is probably a considerable diversity of *Epicephala* remaining to be found. *Epicephala chancapiedra* was associated with herbaceous *Phyllanthus* species belonging to two distantly related subgenera (*Conami* and *Swartziani*). This species is thus likely capable of attacking various herbaceous *Phyllanthus* species and may be found from other herbaceous *Phyllanthus* in other parts of the Neotropics.

Molecular phylogenetic analysis indicated that the four moth species associated with woody *Phyllanthus* are descendants of Old World active pollinators. We observed adult females carrying *Phyllanthus* pollen on the proboscises in all the four species, and pollination in at least two species (*E*. *anomala* and *E*. *graveolensella*). Sensilla on female proboscis were also confirmed in three species (*E*. *acuminatella*, *E*. *graveolensella*, and *E*. *huallagensiella*). These findings indicate that actively pollinating *Epicephala* moths, which originated in the Old World, successfully colonized the New World and remained as active pollinators as they diversified on New World *Phyllanthus*. It is unknown how *Epicephala* moths were able to colonize a remote continent, but a presumably young age of the genus (ca. 25 Ma [12]) suggests that colonization most likely occurred by means of transoceanic dispersal. Simultaneous dispersal of plants and moths (as larvae inside fruits) is unlikely because even if it did occur, emerged adult moths would not survive until the plants become mature enough to flower.

It is, however, not conclusive whether any *Phyllanthus*–*Epicephala* association in the New World is an obligate mutualism (Table 3). In *P*. *graveolens* and *P*. *huallagensis*, flowers that had moth eggs were always pollinated (Table 2), suggesting either that the associated moths pollinate flowers before oviposition, or the moths are capable of distinguishing pollination status and selectively lay eggs in pollinated flowers without pollinating themselves. We consider that the former is more likely because we observed at least one *E*. *graveolensella* actively pollinate prior to oviposition. However, the *P*. *graveolens*–*E*. *graveolensella* association may not be obligate because 42.9% of pollinated flowers did not have moth eggs, indicating that co-pollinators may be present. *Phyllathus huallagensis* may be more dependent on *Epicephala* for pollination because there was a strong association between pollination status and egg load (Table 2), at least in the La Florida population during the period of our fieldwork. The imbricate tepals surrounding the anthers and styles in *P*. *graveolens* and *P*. *huallagensis* resemble those of *Epicephala*-pollinated *Phyllanthus* in the Old World, which hints at the possibility that their flowers are specialized for *Epicephala* pollination. It is also unknown whether there is net positive effect for the plant of being associated with *Epicephala*, because haphazard dissections of fruits with single exit holes indicated that a single moth larva consumes all the six seeds in each fruit in both *P*. *graveolens* and *P*. *huallagensis*. Such a pattern of destructive seed consumption is also the case for *Epicephala*-pollinated *Phyllathus* in New Caledonia, but moth mortality at the immature stage ensures that some seeds remain intact in this case [26]. In any case, actively pollinating *Epicephala* species likely contribute to the pollination of *Phyllanthus* in the New World, but to what extent *Phyllanthus* plants are dependent on *Epicephala* requires further ecological study.

**Table 3:** Summary of the morphological and ecological characteristics of the seven studied *Phyllanthus* species.

Subgenus	Section	Species	Habit	Habitat	Male flower	Female flower	Ecology of associated *Epicephala* moth	Probable pollinator
*Xylophylla*	*Oxalistylis*	*salviifolius*	tree	montane rain forest	tepals flatly open	tepals enfolding pistils	moths oviposit in young fruits but occasionally pollinate actively	*Epicephala* moth (in part)
	*Elutanthos*	*huallagensis*	shrub	seasonally dry forest	tepals enfolding androecium	tepals enfolding pistils	moths actively pollinate and oviposit in flowers	*Epicephala* moth
*Conami*	*Nothoclema*	*acuminatus*	tree	lowland rain forest	tepals flatly open	tepals flatly open	moths oviposit in young fruits	gall midge
		*graveolens*	shrub	seasonally dry forest	tepals enfolding androecium	tepals enfolding pistils	moths actively pollinate and oviposit in flowers	thrips and *Epicephala* moth
	*Apolepis*	*orbiculatus*	herb	lowland rain forest	tepals flatly open	tepals flatly open	moths oviposit in young fruits	ants and other insects
*Swartziani*		*amarus*	herb	lowland rain forest	tepals flatly open	tepals flatly open	moths oviposit in young fruits	ants and other insects
uncertain	uncertain	*stipulatus*	herb	lowland rain forest	tepals flatly open	tepals flatly open	moths oviposit in young fruits	ants and other insects

We also observed the adult behaviors of *E*. *anomala* and *E*. *acuminatella*, but assessing whether they have positive effects on the reproduction of host *Phyllanthus* is not straightforward. Although both species laid eggs in young fruits, and thus most likely did not contribute to pollination, all the female moths that have been examined carried pollen on the proboscises, suggesting that they have the potential to act as pollinators. Because we observed one instance of *E*. *anomala* moth pollinating *P*. *salviifolius* flower (although the flower had been pollinated and started developing into fruit), one possibility is that *E*. *anomala* pollinates only occasionally. The majority of *P*. *salviifolius* female flowers that we observed during the study were already pollinated, but *E*. *anomala* may pollinate more frequently in situations where there are more virgin flowers, although this requires that the moth can discriminate between pollinated and unpollinated flowers. However, *E*. *anomala* lacks the sensilla on the proboscis entirely, which we regard as an indication that the species is evolving toward being parasitic or already is parasitic. The sensilla on the proboscis of *E*. *acuminatella* are also not well developed as compared to other actively pollinating species in Asia (Fig 7) [20], so selection to retain pollination behavior may be relaxed in this species as in *E*. *anomala*. *Epicephala anomala* and *E*. *acuminatella* are closely related on the phylogeny, and thus we hypothesize that there has been a reversal to a less mutualistic lifestyle in the common ancestor of the two species (Fig 8). We did not observe any alternative pollinator in *P*. *salviifolius*, but in *P*. *acuminatus*, gall midges may act as the primary pollinator.

Overall, the finding of *Epicephala* in the New World opens up a new avenue of research on the diversity and evolution of the leafflower–leafflower moth association. A large number of *Epicephala* species is likely to be found throughout the New World, especially in regions of high *Phyllanthus* diversity such as Cuba and Venezuela. *Phyllanthus* species in the sections *Epistylium*, occurring in the Caribbean, and *Microglochidion*, restricted to the tepuis of Guiana Highlands, have non-bifid and fused styles reminiscent of those of *Glochidion* in Asia; thus, obligate mutualistic relationships, as those found in the Old World, may be widespread in some regions or taxonomic groups. On the other hand, some species of New World *Phyllanthus* possess flowers that are most unusual of all *Phyllanthus*, such as *P*. *orbicularis* with large, white, showy tepals, or *P*. *arbuscula* with brightly red flowers borne on flattened, photosynthetic branches (phylloclades). These species likely have entirely different pollination systems, so it is of high interest to clarify how interactions with *Epicephala* and other pollinators have driven the evolution of floral diversity and led to the remarkable radiation of *Phyllanthus* in the New World.

## Supporting information

S1 Fig Mid-point rooted, maximum-likelihood tree of *Epicephala* moths in Peru based on analysis of mitochondrial cytochrome oxidase subunit I (COI) gene sequences. Terminal labels include information on host *Phyllanthus* species name and extraction ID. Numbers above branches are bootstrap values based on 1,000 replications.
(TIFF)

S2 Fig Female and male genitalia of *Epicephala anomala*. (a) Apophyses and eighth abdominal segment; (b) seventh abdominal segment and corpus and ductus bursae; (c) valva; (d) aedeagus. Bar = 500 μm.
(TIFF)

S3 Fig Female and male genitalia of *Epicephala acuminatella*. (a) Seventh abdominal segment and corpus and ductus bursae; (b) Apophyses and eighth abdominal segment; (c) valva; (d) aedeagus. Bar = 500 μm.
(TIFF)

S4 Fig Female and male genitalia of *Epicephala graveolensella*. (a) Apophyses and eighth abdominal segment; (b) seventh abdominal segment and corpus and ductus bursae; (c) valva; (d) aedeagus. Bar = 500 μm.
(TIFF)

S5 Fig Female and male genitalia of *Epicephala huallagensiella*. (a) Apophyses and eighth abdominal segment; (b) seventh abdominal segment and corpus and ductus bursae; (c) valva; (d) aedeagus. Bar = 500 μm.
(TIFF)

S6 Fig Female and male genitalia of *Epicephala chancapiedra*. (a) Female genitalia; (b) valva; (c) aedeagus. Bar = 500 μm.
(TIFF)

S1 Text Descriptions of the five newly described *Epicephala* species. (DOCX)

S1 Video Female *Epicephala anomala* ovipositing into *Phyllanthus salviifolius* ovary without pollinating. (MP4)

S2 Video Female *Epicephala anomala* exhibiting active pollination behavior before attempting to oviposit into *Phyllanthus salviifolius* ovary. Ovipositor of this moth was blocked by the tepal, and oviposition was not successful. The female is the same individual as in S1 Video.
(MP4)
