# Supplementary material for: Leafflower–leafflower moth mutualism in the Neotropics: Successful transoceanic dispersal from the Old World to the New World by actively-pollinating leafflower moths
Source: PLoS One. 2019 Jan 30;14(1):e0210727. doi: 10.1371/journal.pone.0210727 (PMC6353133; doi:10.1371/journal.pone.0210727)
Supplement: S1 Text — (DOCX) [file pone.0210727.s007.docx]

**Species descriptions**

***Epicephala anomala* sp. n.**

Fig. 6; S2 Fig

**Description.** *Wingspan*: 9.0–11.7 mm.

*Head*: With numerous white scales on dorsal surface. Labial palpus with dark brown scales. Antenna brown, about 1.2 × as long as forewing. Female proboscis without trichoid sensilla.

*Thorax*: White dorsally. Forewing brown with narrow white band on dorsum from base to 2/3 of entire length; two pairs of white bands beginning at costal and dorsal margins near 1/3 to 2/3 length of wing and extending obliquely toward wing apex, terminating before reaching mid-width of wing, fading to silver toward wing apex on costal margin; a narrow black intermittent band from 1/2 to 5/6 length near mid-width of wing; a narrow silver band with metallic reflection extending from costa to dorsum at 5/6 length; distal 1/6 orange-brown with black dot centrally, franked by short white spot or band near costa and dorsum; distal end fringed with narrow white band; cilia greyish brown. Hindwing brown, 0.8 × length of forewing; cilia greyish brown.

*Male genitalia*: Tegumen triangular. Cucullus oblong, apex rounded, inner surface covered with numerous hairs; dorsal margin abruptly projecting at 1/6 length, extending inwardly and distally, with numerous spines on surface; ventral margin with sclerotized cone-like projection at 1/5 length and 4 or 5 additional smaller projections from 1/2 to 4/5 length; basal 1/5 of ventral margin attached with a narrow sclerotized blade on posterior side; blade 0.8 × length and 0.7 × width of cucullus, pointed ventro-apically, with membranous sac at joint to ventral margin of cucullus; Sacculus 0.8 × length of cucullus, 2 × width of cucullus near base and tapering at mid-length, rounded apically. Vinculum broad, V-shaped, tapering toward saccus; saccus short. Aedeagus straight, cornutus absent.

*Female genitalia*: Lamella postvaginalis U-shaped, as long as seventh abdominal segment, 2 × width of ostium bursae, stretched outwardly toward distal end. Antrum 0.4 × length of lamella postvaginalis, with bristle of spines on surface. Ductus bursae 2.5 × length of antrum, with weak longitudinal parallel ridges on distal 2/3 length. Corpus bursae oval, as long as ductus bursae; signum absent. Apophyses posteriores about 1.5 × length of apophyses anteriores. Ovipositor dentate laterally, angular at apex.

***Epicephala acuminatella* sp. n.**

Fig. 6; S3 Fig

**Description.** *Wingspan*: 7.0–7.7 mm.

*Head*: With numerous white scales on dorsal surface. Labial palpus with dark brown scales. Antenna brown, about 1.2 × as long as forewing. Female proboscis with trichoid sensilla throughout entire length; sensilla 1/3 to 1/2 width of proboscis.

*Thorax*: White dorsally. Forewing brown with narrow white band on dorsum from base to 1/3 of entire length; two pairs of white bands beginning at costal and dorsal margins near 1/3 to 2/3 length of wing and extending obliquely toward wing apex, terminating before reaching mid-width of wing; a narrow black band from 2/3 to 5/6 length near mid-width of wing; a narrow silver band with metallic reflection extending from costa to dorsum at 5/6 length; distal 1/6 orange-brown with black dot centrally, franked by short white spot or band near costa and dorsum; distal end fringed with narrow white band; cilia greyish brown. Hindwing brown, 0.8 × length of forewing; cilia greyish brown.

*Male genitalia*: Tegumen triangular. Cucullus rectangular oblong, apex rounded, inner surface covered with numerous hairs; dorsal margin abruptly projecting at 1/5 length, extending inwardly and distally, with spines on surface; ventral margin with 7–8 sclerotized cone-like projection from 1/6 to 2/3 length; basal 1/5 of ventral margin attached with narrow, sclerotized, ventrally curved blade on posterior side; blade 0.8 × length and 0.4 × width of cucullus, pointed apically, with membranous sac at joint to ventral margin of cucullus; Sacculus 0.7 × length of cucullus, rectangular, 1.5 × width of cucullus, ventral margin longer than dorsal margin. Vinculum broad, V-shaped, tapering toward saccus; saccus short. Aedeagus straight, cornutus absent.

*Female genitalia*: Lamella postvaginalis V-shaped, about as long as seventh abdominal segment, 4 × width of ostium bursae; distal end of each arm as wide as ostium bursae, jagged. Antrum short, as long as width of ostium bursae, with few sclerotized teeth. Ductus bursae as long as lamella postvaginalis, with a pair of longitudinal parallel ridges for its entire length. Corpus bursae oval, 1.3 × length of ductus bursae; signum absent. Apophyses posteriores about 1.7 × length of apophyses anteriores. Ovipositor dentate laterally, angular at apex.

***Epicephala graveolensella* sp. n.**

Fig. 6; S4 Fig

**Description.** *Wingspan*: 6.7–7.9 mm.

*Head*: With numerous white scales on dorsal surface. Labial palpus with dark brown scales. Antenna brown, about 1.2 × as long as forewing. Female proboscis with trichoid sensilla throughout entire length; sensilla as long as width of proboscis.

*Thorax*: White dorsally. Forewing brown in female and dark brown in male with narrow intermittent white band on dorsum from base to 5/6 of entire length; a pair of white bands beginning at costal margin near 1/3 and 3/5 length of wing and extending obliquely toward wing apex, terminating before reaching mid-width of wing; a narrow silver band with metallic reflection extending from costa to dorsum at 5/6 length; distal 1/6 orange-brown with faint black dot centrally, franked by short white spot or band near costa and dorsum; distal end fringed with narrow white band; cilia greyish brown. Hindwing brown, 0.7 × length of forewing; cilia greyish brown.

*Male genitalia*: Tegumen triangular. Cucullus narrowly oblong, apex rounded, inner surface covered with numerous hairs; ventral half of inner surface with sclerotized projections from base to 1/2 length of cucullus; projections longer toward base of cucullus; base of ventral margin attached with sclerotized, hook-like projection on posterior side. Sacculus 0.7 × length of cucullus, wider than cucullus near base but tapering toward apex, rounded apically. Vinculum broad, V-shaped, tapering toward saccus; saccus short. Aedeagus straight; cornuti consisting of a row of about 10 spines on ventral side from 1/2 to 3/4 length of aedeagus and a slender, weakly curved spine extending from 1/4 to 5/6 length of aedeagus and branching at apex.

*Female genitalia*: Lamella postvaginalis H-shaped, about 0.8 × length of seventh abdominal segment, 1.6 × width of ostium bursae; distal arms shorter and broader than proximal arms, jagged at apex. Antrum short, 1.6 × longer than width of ostium bursae, with bristle of spines on surface. Ductus bursae 2.5 × length of antrum, with weak longitudinal ridges for its entire length. Corpus bursae oval, 0.5 × length of ductus bursae; signum absent. Apophyses posteriores about 2.2 × length of apophyses anteriores. Ovipositor dentate laterally, acute at apex.

***Epicephala huallagensiella* sp. n.**

Fig. 6; S5 Fig

**Description.** *Wingspan*: 8.8–11.2 mm.

*Head*: With numerous white scales on dorsal surface. Labial palpus with dark brown scales. Antenna brown, about 1.2 × as long as forewing. Female proboscis with trichoid sensilla throughout entire length; sensilla 0.5 to 1.0 × as long as width of proboscis.

*Thorax*: White dorsally. Forewing brown in female and dark brown in male with narrow intermittent white band on dorsum from base to 5/6 of entire length; a pair of white bands beginning at costal margin near 1/3 and 3/5 length of wing and extending obliquely toward wing apex, terminating before reaching mid-width of wing; a narrow silver band with metallic reflection extending from costa to dorsum at 5/6 length; distal 1/6 orange-brown with black dot centrally, franked by short white spot or band near costa and dorsum; distal end fringed with narrow white band; cilia greyish brown. Hindwing brown, 0.8 × length of forewing; cilia greyish brown.

*Male genitalia*: Tegumen triangular. Cucullus rectangular oblong, apex rounded, inner surface covered with numerous hairs; proximal 1/4 of ventral margin lined with ventrally projecting hairs. Sacculus 0.8 × length and 1 × width of cucullus, curling longitudinally and inwardly; bilobed at apex; dorsal margin with acute inward projection at 3/5 length and a row of spines from 3/5 length to apex. Vinculum broad, V-shaped, tapering toward saccus; saccus short, 1/2 length of vinculum. Aedeagus straight; cornutus absent.

*Female genitalia*: Lamella postvaginalis widely U-shaped, about 0.4 × length of seventh abdominal segment, 2 × as broad as ostium bursae, jagged at apex. Antrum long, 1.8 × as long as width of ostium bursae. Ductus bursae 2 × length of antrum, with longitudinal parallel ridges on distal half. Corpus bursae oval, 1/2 as long as ductus bursae; signum absent. Apophyses posteriores about 1.8 × length of apophyses anteriores. Ovipositor dentate laterally, angular at apex.

***Epicephala chancapiedra* sp. n.**

Fig. 6; S6 Fig

**Description.** *Wingspan*: 6.3–7.8 mm.

*Head*: With numerous white scales on dorsal surface. Labial palpus with dark brown scales. Antenna brown, about 1.2 × as long as forewing. Female proboscis without trichoid sensilla.

*Thorax*: Greyish brown dorsally. Forewing brown with narrow white band on dorsum from base to 2/3 of entire length; three pairs of white bands beginning at costal and dorsal margins near 1/3 to 3/4 length of wing and extending obliquely toward wing apex, terminating before reaching mid-width of wing; a narrow silver band with metallic reflection extending from costa to dorsum at 5/6 length; distal 1/6 orange-brown with black dot centrally, franked by short white spot or band near costa and dorsum; distal end fringed with narrow white band; cilia greyish brown. Hindwing brown, 0.8 × length of forewing; cilia greyish brown.

*Male genitalia*: Tegumen rounded triangular. Cucullus rectangular oblong, broadened at mid-length, apex rounded, inner surface covered with numerous hairs. Sacculus 0.8 × length and 1 × width of cucullus, acute at apex; distal portion of ventral margin concave. Vinculum U-shaped; saccus slender, 5/6 length of vinculum. Aedeagus straight; dorsal surface with a weakly sclerotized ridge near mid-length; with a sclerotized cone-like projection at 5/6 length.

*Female genitalia*: Lamella postvaginalis forked at mid-length, slightly narrowing toward apex, about 0.7 × length of seventh abdominal segment, 1.2 × as broad as ostium bursae at base. Antrum long, as long as seventh abdominal segment, anterior 1/3 distinctly wider than posterior portion. Ductus bursae 1.3 × length of antrum, with bristle of spines from posterior end to 1/4 of its length and longitudinal parallel ridges from 1/4 to 3/4 length. Corpus bursae oval, as long as combined length of antrum and ductus bursae; signum absent. Apophyses posteriores about 1.8 × length of apophyses anteriores. Ovipositor dentate laterally, acute at apex.
